# Client satisfaction among participants in a randomized trial comparing oral methadone and injectable diacetylmorphine for long-term opioid-dependency

**DOI:** 10.1186/1472-6963-11-174

**Published:** 2011-07-26

**Authors:** Kirsten I Marchand, Eugenia Oviedo-Joekes, Daphne Guh, Suzanne Brissette, David C Marsh, Martin T Schechter

**Affiliations:** 1 Centre for Health Evaluation & Outcome Sciences, Providence Health Care, St. Paul's Hospital 620B - 1081 Burrard Street, Vancouver, BC, V6Z 1Y6, Canada; 2 School of Population and Public Health, University of British Columbia, 2206 East Mall Vancouver, BC, V6T 1Z3, Canada; 3 Centre de Recherche du Centre Hospitalier de l'Université de Montréal (CHUM), 1058 St-Denis Montréal, QC, H2X 3J4 Canada; 4 Centre for Addiction Research BC, University of Victoria, 2300 McKenzie Ave, Victoria, BC, V8P 5C2, Canada; 5 Northern Ontario School of Medicine, 935 Ramsey Lake Road, Sudbury, ON, P3E 2C6, Canada

## Abstract

**Background:**

Substitution with opioid-agonists (e.g., methadone) has shown to be an effective treatment for chronic long-term opioid dependency. Patient satisfaction with treatment has been associated with improved addiction treatment outcomes. However, there is a paucity of studies evaluating patients' satisfaction with Opioid Substitution Treatment (OST). In the present study, participants' satisfaction with OST was evaluated at 3 and 12 months. We sought to test the relationship between satisfaction and patients' characteristics, the treatment modality received and treatment outcomes.

**Methods:**

Data from a randomized controlled trial, the North American Opiate Medication Initiative (NAOMI), conducted in Vancouver and Montreal (Canada) between 2005-2008, was analyzed. The NAOMI study compared the effectiveness of oral methadone *vs*. injectable diacetylmorphine over 12 months. A small sub-group of patients received injectable hydromorphone on a double blind basis with diacetylmorphine. The Client Satisfaction Questionnaire (CSQ-8) was used to measure satisfaction with treatment. CSQ-8 scores, as well as retention and response to treatment, did not differ between those receiving hydromorphone and diacetylmorphine at 3 or 12 months assessments; therefore, these two groups were analyzed together as the 'injectable' treatment group.

**Results:**

A total of 232 (92%) and 237 (94%) participants completed the CSQ-8 at 3 and 12 months, respectively. Participants in both groups were highly satisfied with treatment. Independent of treatment group, participants satisfied with treatment at 3 months were more likely to be retained at 12 months. Multivariate analysis indicated that satisfaction was greater among those randomized to the injection group after controlling for treatment effectiveness. Participants who were retained, responded to treatment, and had fewer psychological symptoms were more satisfied with treatment. Finally, open-ended comments were made by 149 (60.3%) participants; concerns about the randomization process and the study ending were most commonly reported by participants receiving the oral and injectable medications, respectively.

**Conclusions:**

The higher satisfaction among those receiving medically prescribed injectable diacetylmorphine (or hydromorphone) supports current evidence regarding the attractiveness of this treatment for long-term, opioid-dependent individuals not benefiting sufficiently from other treatments. In addition, the measurement of treatment satisfaction provides valuable information about participants at risk of relapse and in need of additional services.

**Trial Registration:**

ClinicalTrials.gov Identifier: NCT00175357

## Background

Opioid dependence is a chronic relapsing disease that when untreated can lead to moderate to severe health consequences such as blood-borne viral infections, endocarditis, and drug overdoses [1,2]. Treatment for opioid dependence can be focused on achieving abstinence of any drugs or aimed at reducing the adverse health consequences associated with opioid dependence. Also, delivery of opioid dependence treatment can be short or long-term and at in or out-patient settings. Psychotherapy and counselling can be provided alone or in combination with other treatments [3-5].

Substitution with opioid-agonists (e.g., methadone) is a long-term treatment aimed at reducing the use of illicit opioids and its associated problems, including reduced illicit drug use and illegal activities, human immunodeficiency virus (HIV) infections, as well as improved general health and psychosocial adjustment [2,3,6]. In addition, patients on Opioid Substitution Treatment (OST) are effectively retained [7], and studies have shown that the time patients remain in treatment is associated with greater improvements in the above mentioned areas [8]. The delivery of OST varies widely, even in the same region, in its setting, philosophy, availability of ancillary services, and policies. Several manuals and guidelines for best practices have been developed for health care providers as well as for specific professional roles [9,10].

It is common for health care services to routinely assess patient's satisfaction with received treatments in order to gain feedback which can be used to improve the services provided. Satisfaction with received care can be used as a measure of quality of care and perceived benefits of a service [11,12]; however, this is a multi-dimensional concept and it is affected by a wide range of factors. For instance, satisfaction may be influenced by the characteristics of the patient (e.g., expectations, demographic characteristics, beliefs, previous treatment experiences, etc.), the illness being treated (e.g., acute *vs*. chronic), the treatment modalities being used (e.g., inpatient *vs*. outpatient), and the characteristics of the individual providing the treatment (e.g., authoritarian, tolerant, etc.) [11-16].

Client satisfaction has been evaluated in addiction treatment services, including OST, though, to a much lesser degree than other areas of care [17-20]. Although patients tend to report high levels of satisfaction with addiction treatment [12,13,15,21,22], patients receiving OST have also expressed concerns related to the demands of this treatment. For example, concerns regarding the procedures of clinics, including dispensing schedules [20], prolonged waiting times and lack of access to ancillary services [23] have been perceived as having an impact on patients' satisfaction with OST. Moreover, Perez de los Cobos et al., [18] reported that the number of hours per week that methadone was dispensed was independently associated with treatment satisfaction, and a later study found that patients' who had a high regard of methadone as a medication for treating opioid dependence were more satisfied [19].

Client satisfaction has also been linked with a number of favourable addiction treatment outcomes, including higher service use [15,24], reduction in substance use [15,24-27], greater physical and mental health [25], psychosocial improvements [26], and greater retention in treatment [12,16,26]. For example, Kelly et al. [26] recently determined a negative association between patients' treatment satisfaction and illicit drug use and illegal activity at 3 months after OST initiation and a positive association with treatment retention at 12 months. In contrast, in an outpatient alcohol and drug treatment setting that provided intensive treatment by patient education, group therapy, and social support, McLellan & Hunkeler [28] did not find an association between patient satisfaction and treatment outcomes at 6 months follow-up.

Between 2005 and 2008 a randomized controlled trial (RCT) comparing the effectiveness of two OST modalities for the treatment of long-term, treatment-resistant opioid dependence, was carried out in Vancouver and Montreal (Canada). This study analyses data from that trial with the purpose to: a) determine participants' satisfaction with the treatment received; and b) test if satisfaction scores vary according to patients' characteristics, the treatment modality received and treatment outcomes.

## Methods

### Design, Setting and Participants

The North American Opiate Medication Initiative (NAOMI) was an open-label, phase III RCT comparing supervised injectable diacetylmorphine and oral methadone in the treatment of long-term opioid dependence. For the purpose of validating self-reported use of street heroin by urine testing, a small group of participants were randomized to receive injectable hydromorphone in double-blind basis instead of diacetylmorphine. Participants were aged 25 or greater, with at least 5 years of opioid dependence (according to DSM-IV criteria), current daily injection of opioids, a minimum of two previous treatments for opioid dependence, including at least one OST attempt and no enrolment in OST within the prior 6 months. Data regarding participants demographics, substance use before and during the study, dosage and monitoring issues have been published elsewhere [29-31]. Briefly, at treatment entry, participants in both sites were deemed poly drug users, with heroin and cocaine being the two most frequent drugs reported in lifetime and prior month. Comparisons between Vancouver and Montreal showed that a higher proportion of Vancouver participants were Aboriginal (31.6% *vs*. 0.0%), had unstable housing (88.5% vs. 22.0%) and had more days of smoked crack cocaine use (16.88 *vs*. 2.25) in relation to Montreal [31]. During treatment, illicit heroin use declined drastically among those randomized to injectable diacetylmorphine (from 26.6 to 5.3 days), while days of cocaine use remained stable, as per intention-to-treat (ITT) analysis [29]. After excluding each participant's initial 90 day dose adjustment, the average dose during the study period for participants receiving diacetylmorphine, hydromorphone, or methadone was 395.8 mg, 186.7 mg and 96.0 mg, respectively [32]. High-performance liquid chromatography testing was used to detect the presence of morphine and 6-monoacetylmorphine, as evidence of illicit heroin use in urine samples of the methadone and hydromorphone groups, which showed a significantly higher use of illicit heroin in the methadone compared to hydromorphone group [33].

A total of 251 individuals were randomized to receive either oral methadone (n = 111), injectable diacetylmorphine (n = 115) or injectable hydromorphone (n = 25), the latter two on a double blind basis. Participants administered the injection medications up to three times daily (up to 1,000 mg per day) under the supervision of clinic staff; oral methadone was dispensed daily.

Participants were offered psychosocial and primary care; in keeping with Health Canada Best Practices, all services were delivered in a patient-centred fashion [9]. Participants received their allocated treatment for 12 months. Since the injectable medications are not licensed in Canada for the treatment of opioid-dependency, an additional 3-month period was provided to taper and transition those participants to other treatment modalities (primarily methadone). The University of British Columbia/Providence Health Care research ethics board approved the study, and all participants provided written informed consent.

### Measures

A separate research team obtained outcome evaluations at baseline and follow-up (3, 6, 9 and 12 months), using the European Addiction Severity Index (EuropASI; [34]) and Maudsley Addiction Profile (MAP; [35]). Participants were considered retained to addiction treatment if they received study medication on at least 10 out of the 14 days prior to the follow-up assessment or were confirmed to be in any other treatment program or abstinent of opioids during this interval. Participants were regarded as treatment compliant if they received the treatment they were allocated to for at least 20 out of the prior 30 days (i.e., those that were randomized to injectable medication continued to receive injectable; those randomized to optimised methadone were still receiving methadone at the study clinic). Overall clinical response was defined as an improvement of at least 20% in Drug and/or Legal EuropASI composite scores, with no deterioration higher than 10% in more than one of the remaining composite scores. All participants lost to follow-up were considered non-retained.

Participants' satisfaction with treatment was evaluated at 3 and 12 months using the Client Satisfaction Questionnaire (CSQ; [36], 8-item version [37]. The CSQ-8 assesses global patient satisfaction with a 4-point Likert type scale and also provides a general score ranging from 8 to 32. This questionnaire has been used in psychiatric inpatient and outpatient treatment evaluations [38,39] as well as in substance abuse treatment settings [40], including OST [21,22,41-44]. The CSQ-8 includes the following questions: 1) How would you rate the quality of service received?; 2) Did you get the kind of service you wanted?; 3) To what extent has our programme met your needs?; 4) If a friend were in need of similar help, would you recommend our programme to him or her?; 5) How satisfied are you with the amount of help you have received?; 6) Have the services you received helped you to deal more effectively with your problems?; 7) In an overall, general sense, how satisfied are you with the service you have received?; 8) If you were to seek help again, would you come back to our programme? At the end of the questionnaire participants had the option to make comments or suggestions.

### Analysis

Item and total mean treatment satisfaction scores were calculated for each participant and to obtain a global satisfaction score. The groups receiving hydromorphone and diacetylmorphine did not differ at 3 or 12 months assessments in their CSQ-8 scores; therefore, these two groups were analyzed together as the 'injectable' treatment group. Comparisons of client satisfaction between treatment groups (injectable diacetylmorphine or hydromorphone *vs*. oral methadone) were carried out using Student's t and Mann-Whitney U depending on variable distribution, as well as for retention in addiction treatment and clinical response.

Similar to other studies [12,13,15], participants scored very high on treatment satisfaction, and as a consequence the variable's distribution was skewed. To fit into the multivariate models, mean satisfaction scores were organized according to 3 levels of satisfaction: 1) dissatisfied or least satisfied (8-16); 2) satisfied (17-30); and 3) very satisfied (31-32). Satisfaction scores were evaluated in relation to patient characteristics (age, gender, site, ethnicity) as well as their scores at baseline, 3 and 12 months follow-up (EuropASI; MAP). A multivariate logistic regression analysis, adjusted by treatment group, was used to determine if total and each item of the client satisfaction questionnaire at 3 months predicted treatment outcomes at 12 months. Finally, a multivariate proportional odds model estimated by Generalized Estimating Equation (GEE) algorithm was performed to determine predictors of treatment satisfaction for total score and each individual question. Randomization group, gender, ethnicity, age, and site were included in the model whether significant or not. All other variables remained in the model only if significant. Missing values were considered as missing. Analyses were performed by an ITT basis. Data was analyzed using SAS^® ^(version 9.1.3).

The open-ended qualitative responses provided in the CSQ-8 'comments and suggestions' section were also analysed. To start, two researchers read all comments (EOJ; KM) and an initial list of themes were developed. The researchers then independently coded each comment according to these themes. Finally, the researchers decided on a list of 12 common categories based on the frequency which participants mentioned them: positive or negative comments about the staff and the program, desire that study eligibility criteria were easier, frustration with the randomization process, dissatisfaction with medication dose received, lack of attention to their pain management, extension of clinic operation hours, request for more ancillary services, request of nutritional services, concerns regarding the interactions in the clinic while waiting for the medication, lengthy pre-post injection assessment, insufficient time allotted to inject, and disappointments of the study ending. A univariate logistic GEE model was applied to find subgroups of participants most likely to make open-ended comments at 3 and 12 months follow-up.

## Results

A total of 88 (91%) women and 144 (93%) men completed the CSQ-8 at 3 months; at 12 months, 91 (94%) women and 146 (95%) men completed the CSQ-8. Compared to participants in the oral methadone group, those randomized to the injection group had a significantly higher total satisfaction score at both 3 and 12 months (Table 1). These participants also showed significantly higher satisfaction scores for CSQ item 3 (program meeting their needs), item 6 (the services helped them to deal with their problems) and item 8 (willingness to return for treatment in the future).

**Table 1:** Treatment Satisfaction by treatment group and time

	T3				T12			
**Treatment Satisfaction Questions **^a^	**Oral**		**Injection**		**Oral**		**Injection**	

	**M**	**SD**	**M**	**SD**	**M**	**SD**	**M**	**SD**

Quality of service received	3.2	0.95	3.4	0.76	3.3	0.85	3.3	0.86

Get the kind of service wanted^b^	2.7	1.15	3.5	0.64	3.0	1.11	3.2	0.86

The program met the needs^b; c^	2.4	1.03	3.2	0.70	2.7	1.02	3.2	0.83

Would recommend the program^b^	3.3	0.96	3.8	0.45	3.3	0.93	3.6	0.84

Satisfied with the amount of help received^b^	2.9	0.97	3.4	0.67	3.1	0.95	3.2	0.89

The services helped to deal with problems^b; c^	2.8	0.95	3.6	0.63	3.0	0.88	3.3	0.80

General satisfaction with the service received^b^	2.9	1.02	3.4	0.70	3.1	0.91	3.2	0.92

Would come back^b; c^	3.2	0.92	3.7	0.53	3.4	0.86	3.6	0.68

Total Satisfaction^b; c^	23.6	6.41	28.0	3.78	24.8	6.00	26.5	5.28

^a ^Refers to questions and total score from CSQ-8

^b ^Differences between groups significant (p < 0.01) at 3 months

^c ^Differences between groups significant (p < 0.04) at 12 months.

After adjusting for treatment group, the CSQ item 3 (to what extent has our programme met your needs) at 3 months significantly predicted retention outcomes at 12 months. Compared to participants who answered that none or few of their needs had been met, those most satisfied and satisfied at 3 months, were more likely to be retained to addiction treatment at 12 months (most satisfied Odds Ratio [OR] = 6.2; 95% Confidence Interval [CI] = 2.23, 17.39; p = 0.001; satisfied OR = 3.3; 95% CI = 1.5-7.1; p = 0.003). Similar results were obtained for retention to allocated treatment; however, 3 month satisfaction did not predict 12 month treatment response.

Table 2 displays the multivariate GEE analysis for total satisfaction and each item score of the CSQ-8. Randomization to the injectable group, younger age, better psychological health, and less drug use predicted treatment satisfaction in most of the scores. Compared to women, and non-Aboriginal participants, men and Aboriginal participants tend to be less satisfied. Participants who were compliant, retained and responded to treatment also tended to be more satisfied.

**Table 2:** Multivariate Generalized Estimating Equation model for predictors of treatment satisfaction

Variable	**TotalS **^**i †**^	**Q1**^**i †**^	**Q2**^**i †**^	**Q3 **^**i †**^	**Q4**^**i †**^	**Q5**^**i †**^	**Q6**^**i †**^	**Q7 **^**i †**^	**Q8 **^**i †**^
Randomization group: Injectable *vs*. Oral	1.32 (0.81-2.16)	1.05 (0.74-1.50)	**1.81** **(1.26-2.59)**	**2.40** **(1.61-3.56)**	**1.94** **(1.26-2.98)**	1.14 (0.78-1.67)	**2.35** **(1.62-3.41)**	1.16 (0.79-1.72)	**2.29** **(1.51-3.48)**

Gender: Male vs. Female	0.65 (0.41-1.03)	0.84 (0.58-1.21)	0.75 (0.52-1.07)	0.82 (0.56-1.20)	**0.55** **(0.36-0.86)**	0.74 (0.51-1.09)	0.74 (0.51-1.08)	0.86 (0.60-1.25)	**0.63** **(0.42-0.96)**

Age	**0.71** **(0.54-0.95)**	0.99 (0.80-1.22)	0.81 (0.65-1.00)	**0.69** **(0.55-0.86)**	**0.68** **(0.52-0.88)**	**0.74** **(0.58-0.93)**	0.79 (0.63-1.00)	**0.75** **(0.60-0.94)**	**0.78** **(0.60-1.00)**

Ethnicity: Aboriginal *vs*. Non-Aboriginal	0.70 (0.41-1.20)	0.80 (0.53-1.22)	0.76 (0.50-1.15)	0.99 (0.65-1.49)	**0.55** **(0.34-0.89)**	1.01 (0.67-1.54)	0.99 (0.63-1.55)	0.72 (0.47-1.11)	**0.50** **(0.31-0.81)**

Site: Vancouver *vs*. Montreal	0.97 (0.54-1.74)	0.72 (0.45-1.16)	1.51 (0.98-2.33)	1.31 (0.82-2.10)	**2.16** **(1.31-3.54)**	1.19 (0.75-1.88)	1.07 (0.68-1.69)	1.00 (0.63-1.58)	**1.74** **(1.06-2.84)**

Treatment Compliance:^a^ Yes *vs*. No	**1.80** **(1.08-3.01)**	-	**2.18** **(1.51-3.12)**	-	**2.44** **(1.60-3.72)**	**1.97** **(1.37-2.86)**	**2.10** **(1.45-3.03)**	-	**1.95** **(1.29-2.94)**

Treatment Response:^b^ Yes *vs*. No	-	-	**1.53** **(1.09-2.13)**	-	-	**1.52** **(1.06-2.17)**	-	-	

Treatment Retention:^c^ Yes *vs*. No	-	-	-	**2.31** **(1.41-3.80)**	-	-	-	**2.15** **(1.36-3.40)**	

Current Psychological Symptoms^d^	**0.94** **(0.91-0.97)**	-	**0.95** **(0.93-0.97)**	**0.95** **(0.93-0.97)**	**0.95** **(0.93-0.97)**	**0.96** **(0.94-0.98)**	**0.95 (0.93-0.97)**	**0.95 (0.93-0.97)**	**0.95 (0.92-0.97)**

Current Physical Health^d^	-	**0.95** **(0.93-0.97)**	-	-	-	-	-	-	-

Current Legal Situation^e^	-	-	**-**	**0.92** **(0.85-0.99)**	-	-	-	-	-

Current Drug Use^e^	**0.81** **(0.72-0.92)**	**0.96** **(0.93-0.99)**	**-**	**0.79** **(0.71-0.87)**	**0.89** **(0.80-0.99)**	**0.90** **(0.82-1.00)**	**0.85** **(0.78-0.94)**	**0.81** **(0.74-0.89)**	-

Family/Social Relations^e^	-	-	-	-	-	**-**	-	-	**0.90** **(0.82-0.99)**

Heroin Use at Baseline^f^	-	-	-	-	-	**0.96** **(0.94-0.99)**	-	-	-

**^† ^**Refers to CSQ-8 (TotalS: Total Satisfaction score; Q1: How would you rate the quality of service received; Q2: Did you get the kind of service you wanted; Q3: To what extent has our program met your needs; Q4: If a friend were in need of similar help, would you recommend our program to him or her; Q5: How satisfied are you with the amount of help you have received; Q6: Have the services you received helped you to deal more effectively with your problems; Q7: In an overall, general sense, how satisfied are you with the service you have received; Q8: If you were to seek help again, would you come back to our program?)

**^i ^**Odds Ratio (95% Confidence Interval). P values highlighted: p ≤ 0.05

(a) Retention to allocated treatment: at least 20 out of prior 30 days;

(b) Treatment response defined as improvement of at least 20% in Drug and/or Legal EuropASI composite scores, with no deterioration higher than 10% in more than one of the remaining composite scores;

(c) Retention to addiction treatment or abstinent: at least 10 out of prior 14 days;

(d) MAP (Maudsley Addiction Profile) in the prior month. Scores range from 0 to 40; higher scores are indicative of more severe problems. Higher scores are indicative of more severe problems;

(e) EuropASI (European version of the Addiction Severity Index) in the prior month. Sub-scale scores range from 0 to 1; higher scores are indicative of more severe problems;

(f) In the prior 30 days.

Open-ended comments about treatment satisfaction and perceptions were provided by 149 (60.3%) of the participants. Participants who were older (OR = 1.5; 95% CI = 1.2- 1.9; p = 0.002), interviewed in Vancouver (OR = 6.8; 95% CI = 3.9- 12.0; p = < 0.0001), and who were receiving their allocated treatment in the prior month (OR = 6.8; 95% CI = 3.9-12.0; p = 0.0251) were significantly more likely to make open-ended comments. Table 3 shows that general comments about staff and the program were made by 43 (38.7%) and 54 (58.7%) participants at 3 months and at 12 months, respectively. Concerns about randomization were mostly mentioned at 3 months compared to the 12 month evaluation, while disappointments about the end of study were mentioned more frequently at 12 months. When comparing comments made by oral (n = 86) and injectable (n = 120) treatment groups, individuals in the latter group made more general comments about the staff and program and these also tended to be more positive. Only participants receiving the oral medication expressed their frustration with the randomization process, while disappointments about the study ending were mentioned more by those in the injection group.

**Table 3:** Categories of comments by group

**Comments **^**a**^	Oral n = 86		Injection n = 120		Total n = 206	
	**n**	**(%)**	**n**	**(%)**	**n**	**(%)**

Comments about staff and program	28	32.6	69	57.5	97	47.1

Positive	24	27.9	54	45.0	78	80.4

Negative	4	4.7	15	12.5	19	19.6

Easier eligibility	5	5.8	4	3.3	9	4.4

Dissatisfaction with Dosage	1	1.2	7	5.8	8	3.9

Frustration with Randomization	31	36.0	0	0.0	31	15.0

Insufficient Pain Management	3	3.5	1	0.8	4	1.9

Extension of Clinic Operation Hours	8	9.3	4	3.3	12	5.8

Request More Ancillary services	3	3.5	5	4.2	8	3.9

Request More Nutritional services	3	3.5	5	4.2	8	3.9

Concerns of Interactions in the Waiting Room	0	0.0	8	6.7	8	3.9

Lengthy Pre-Post Injection Assessment	0	0.0	8	6.7	8	3.9

Insufficient Time Allotted to Inject	0	0.0	4	3.3	4	1.9

Disappointments of the Study Ending	3	3.5	20	16.7	23	11.2

^a ^All comments were derived from the open-ended remarks section of the CSQ-8. Comments were not mandatory.

## Discussion

The present study determined participants' satisfaction with received treatments in the first North American RCT to provide injectable diacetylmorphine or hydromorphone compared to oral methadone for the treatment of long-term, treatment resistant, opioid-dependency. At 3 and 12 months, participants were satisfied with the treatment received during the study period, although satisfaction was greater for those randomized to receive injectable treatments. At 3 months, participants who reported that the program met their needs were more likely to be retained at 12 months. To our knowledge this is the first study to assess treatment satisfaction among participants receiving supervised injectable diacetylmorphine or hydromorphone.

Regardless of the outcome of the randomization, participants in the trial were highly satisfied with the treatment received. This follows previous studies which have consistently found that patients tend to report high levels of treatment satisfaction, including community health services [45], services for mental health [13], addiction [46], and opioid dependence [20]. In a meta-analytic review of patient satisfaction with mental health services, it was suggested that the high skewness towards positive satisfaction scores might be due to the lack of clarity of norms against which to compare the treatments, the psychometric properties of the instruments used, and the lack of a specific theoretical framework of patient satisfaction [13]. The factor structure (uni-dimensional *vs*. multi-dimensional) of an instrument can also have an impact on the sensitivity and validity of the instrument's ability to measure satisfaction [47]; therefore, uni-dimensional instruments might overestimate participants' satisfaction because participants are dichotomously categorized as being either satisfied or dissatisfied. More recently, in a European review of outpatient care, Saila et al. [12] suggested that the generally high levels of satisfaction may also reflect a publication bias, where researchers are more likely to publish findings that are favourable to their particular programs or services. Another hypothesis to consider is that an ill person who receives care will most likely be grateful to the caregiver, adding to the positive skewness in satisfaction scores found in the literature as well as in this study.

In spite of being highly satisfied, participants who reported that the program met their needs at three-months were more likely to be retained in treatment at 12 months. Several other studies have reported a similar association between satisfaction and retention to addiction treatment services [12,26,41,46]. In particular, Kelly et al. [26] recently found that those participants who were retained in OST at 12 months were more satisfied with the program at 3 months. Therefore, the integration of client satisfaction surveys early in the treatment program may be a useful tool to identify participants who are at risk for treatment dropout and relapse.

In the present study, participants randomized to receive injectable medications were significantly more satisfied than those randomized to oral methadone even after controlling for age, gender, ethnicity, treatment retention, treatment compliance and treatment response. Furthermore, differences in satisfaction scores between treatment groups were not the result of patient characteristics at baseline (e.g., age, gender, education, drug use severity, health). To date, there is very limited data on treatment satisfaction among opioid-dependent individuals receiving injectable OST. In the only study (to our knowledge) that assessed client satisfaction among patients receiving (in the UK) injectable OST (diacetylmorphine or methadone), those receiving diacetylmorphine were more satisfied, indicating the treatment was more beneficial because of the ease of injecting diacetylmorphine as well as their preference for this drug over methadone [20]. The higher client satisfaction scores among participants receiving injectable diacetylmorphine (or hydromorphone) indicate that this is a treatment that long-term opioid-dependent individuals would like to receive. This is highly relevant when considering that these individuals are often difficult to engage and retain in treatment, and when untreated are at greater risk for mortality and morbidity [1,2,48].

In our multivariate analysis, older age was strongly associated with treatment satisfaction. In addition, men and Aboriginal participants were less likely to recommend the program to friends or relatives (CSQ-Q4) and to respond that they would return in the future for a similar program (CSQ-Q8). Studies in psychiatric and prison based settings have found older participants to be more satisfied with treatment [49,50], and it was suggested that older patients may be more adaptable, respectful, and have lower expectations as a result of having more previous treatment experiences. In contrast, younger patients have fewer previous experiences with which to compare their treatment, and may be more defiant toward other patients and staff. The association between gender and treatment satisfaction is less consistent; however, in one review of treatment satisfaction with general health care services, it was suggested that women and older patients tend to be more satisfied with treatment [51]. Regarding ethnicity, there is a paucity of data reporting an association between treatment satisfaction and participants' ethnicity. In their review, Aharony et al. [51] briefly mention one study that found lower satisfaction among African American participants compared to Caucasian participants. Therefore, given the state of the current literature it is difficult to interpret our findings regarding the association between socio-demographic factors and treatment satisfaction. To our knowledge this is the first study that has considered Aboriginal ethnicity in the evaluation of treatment satisfaction with OST in Canada. Previous analyses of this study data have demonstrated no differences in treatment effectiveness by ethnicity [52]. However, in our multivariate model that controls for treatment effectiveness, Aboriginal participants were less satisfied with the treatment program. These findings are highly relevant when considering that there is data indicating that Aboriginal people are less likely to access methadone maintenance treatment [52,53] and face specific barriers [54]. Treatment satisfaction might be a useful tool to identify treatment needs among Aboriginal people with chronic long-term opioid dependence.

Treatment compliance, response, retention and drug use have previously been associated with treatment satisfaction [12]. For example, studies have found an association between client satisfaction and a reduction in substance use during [26] and after the treatment period [25,27,55,56]. Therefore, the results obtained in our study, where lower drug use, higher retention, response and compliance with treatment were positively associated with treatment satisfaction, were expected.

Satisfaction with treatment in the present study was also more likely among those with better psychological status. In a recent study, Hser et al. [46] found that participants with severe psychological and physical health symptoms at treatment initiation were more satisfied when provided with additional intense services. Moreover, Kelly et al. [26] found that patients with the greatest treatment needs had more psychological problems. Together, these results suggest that unmet treatment needs may be greater among participants with worse psychological health, which may contribute to lower satisfaction with the services provided. Thus, satisfaction with treatment may be an indicator of participants' treatment needs.

In the open-ended comments sections, participants in the oral group expressed their concern with the randomization process, while those in the injection group were more worried about the study ending. It is well known that in clinical trials, randomization to the less desired treatment condition (e.g., placebo, treatment as usual) might have a significant impact on participants willingness to adhere and comply with the study protocol; this is especially true in drug and alcohol treatment settings where attendance is already low [57]. Reviews of recruitment in RCTs have established that one of the most common concerns participants express is their uncertainty with the study medications and not being randomized to their preferred treatment [58,59]. As a result, participants are often dissatisfied with their treatment allocation [59]. Although participants in this study understood the *probability *of being randomized to the oral group, the *desired *treatment condition was injectable diacetylmorphine. Thus, it is critical to consider and assess how the randomization process impacts participants, and highlight the need of the provision of additional supports for participants and recruitment staff in this phase of a study.

In addition to common concerns among participants in RCTs, comments regarding the provision of OST were also expressed in the present study. Clinic operating hours, as an aspect of the treatment delivery that could be improved, were mentioned in participants' comments, and mostly by those in the oral treatment group. This result is consistent with a previous study of treatment satisfaction among patients receiving MMT, where the number of hours per week that methadone was dispensed was the only variable that predicted client satisfaction [18]. Comments that were specific to those in the injectable group included interactions in the waiting room, the lengthy time required for pre- and post-injection assessments as well as insufficient time permitted in the injection room. It is well known that the demands of injectable OST are much greater for both patients and staff; patients receiving injectable treatment in the NAOMI study attended the clinic up to 3 times daily, for approximately 1 hour each visit. Considering the benefits associated with injectable treatments and the overall greater satisfaction among patients receiving this form of treatment, these issues are being further explored with both patients and staff for future studies.

Limitations derived from the design of the parent study have been discussed elsewhere [29]. It is important to note that the NAOMI sample is a very homogenous group of opioid-dependent individuals with several previous drug treatment attempts that have been injecting drugs for an average of 15 years and were not benefiting from the available addiction treatment system. Also, for our theoretical framework regarding the parameters from which treatment satisfaction is being compared in this study, it is important to consider that: a) participants were not receiving any treatment for at least six months before randomization; b) most participants enrolled into this treatment hoping to receive injectable medications; c) the 'methadone' group never received injectable medications, however the injectable group (as well as the oral group) did in the past receive oral methadone (the gold standard to compare with); and d) the unidimensional factor structure of the CSQ-8 may have yielded an overestimation of satisfaction. A 'social desirability bias' (reply in a manner that will be viewed favourably by others) may also have influenced some participants' responses, accounting for the positive responses.

## Conclusions

Among long-term chronic opioid injectors participating in a randomized clinical trial prescribing injectable diacetylmorphine or hydromorphone and oral methadone, those receiving injectable medications were more satisfied with treatment. Independent of treatment group, treatment satisfaction was also an indicator of retention in treatment, as well as treatment response, including a reduction in substance use. As the first study in North America to provide injectable OST, these findings have valuable implications for future RCTs, which should continue to measure satisfaction in order to identify areas of improvement. These findings also provide evidence-based knowledge for good clinical practice guidelines in the treatment of chronic opioid dependence in Canada as they highlight the association between treatment satisfaction and improved treatment outcomes, particularly for those receiving more innovative treatment medications.

## Author Disclosures

### Role of Funding Source

The study was funded by the Canadian Institutes of Health Research (CIHR). CIHR had no further role in study design; in the collection, analysis and interpretation of data; in the writing of the report; or in the decision to submit the paper for publication.

## Competing interests

The authors declare that they have no competing interests.

## Authors' contributions

MTS, SB, DM made substantial contributions to conception and design of the parent study; MTS, SB, DM, EOJ and DG, made substantial contributions to acquisition of data, and analysis and interpretation of data; KM made substantial contributions to analysis and interpretation of data. The first (KM), second (EOJ) and last author (MTS) wrote the first draft of the paper, the senior statistician (DG) performed the data analyses. All authors critically revised the manuscript for important intellectual content. The final decision about publishing the paper was made by all the authors. All authors read and approved the final manuscript.

## Pre-publication history

The pre-publication history for this paper can be accessed here:

http://www.biomedcentral.com/1472-6963/11/174/prepub
